# Reinforcing kangaroo mother care uptake in resource limited settings

**DOI:** 10.1186/s40748-018-0091-3

**Published:** 2018-12-04

**Authors:** Subhashchandra Daga

**Affiliations:** Pacific Medical College and Hospital, Udaipur, 313 001 India

**Keywords:** Kangaroo-mother-care, Skin-to-skin-contact, Keeping babies warm, Thermal control of the newborns, Hypothermia in a newborn

## Abstract

**Background:**

The national and global coverage of kangaroo mother care (KMC) remains low. Hence, adjuncts to KMC may be necessary, especially on day1 of life when neonatal mortality is high. It is important to provide warmth and reduce mortality in preterm low birth weight (LBW) infants in the community/hospital setting. In this manuscript, the outcome of using a Styrofoam box (SB) for LBW infants in various situations in India, such as in a home-setting in tribal/extra-remote areas, at a primary health center in tribal/extra-remote areas and at a referral hospital, is presented. It is suggested that use of an SB may complement KMC.

**The study:**

In this retrospective observational study, an SB (50 × 36 × 25 cm, weight: 500 g) was used in diverse settings: a) as a home incubator in the early neonatal period, b) for providing warmth after hospital discharge and c) as a transport incubator for home-to-hospital and inter-hospital transportation.

a) All six infants, presenting on day 1 of life with a foot length of less than 6.5 cm, remained warm and survived when the box was used as a home incubator. b) The babies discharged from hospital (*N* = 7) were warm in the box at the home setting. c) Use of the box as a home-to-hospital transport incubator improved the number of referrals from 13 to 24 in one year. d) Oxygen saturations were well-maintained and hypothermia did not occur in any infant during inter-hospital transfers when oxygen was administered in the SB. e) The concentration of oxygen delivered was predictable and was well maintained when administered to infants in the SB. The acceptance of the use of an SB by the parents was beneficial.

**Conclusion:**

An SB may be used to complement KMC in resource-limited settings. Well-designed studies are required to confirm the safety and efficacy of this approach in reducing neonatal hypothermia, morbidity, and mortality.

## Background

In a review of 21 randomized controlled trials (3042 infants), the researchers compared conventional neonatal care with kangaroo mother care (KMC). They found that KMC reduced mortality and severe infection/sepsis, nosocomial infection/sepsis, hypothermia, severe illness, and lower respiratory tract disease. They also observed that the growth parameters were superior in the KMC group. The authors concluded that KMC is an effective and safe alternative to conventional neonatal care for low birth weight (LBW) infants, mainly in resource-limited countries [1]. However, a recent study conducted at a tertiary care center in Zambia observed that the proportion of neonates with moderate or severe hypothermia did not differ between the KMC and control groups at 1 h after birth or at discharge [2]. Despite overwhelming evidence in favor of KMC, the national and global coverage of KMC remains low [3]. Therefore, adjuncts to KMC may be necessary, especially on day 1 of life when neonatal mortality is high. It is worth noting that few studies of KMC enrolled infants on day 1 after birth. Styrofoam boxes (SBs) have been used as home incubators during the early neonatal period, during home-to-hospital and inter-hospital transportation, and for providing warmth after hospital discharge.

This manuscript proposes the use of SB to complement KMC by keeping the baby warm. The strategy may be to keep the baby in an SB when the baby is not in KMC.

This manuscript describes/reviews previous SB investigations in the urban as well as rural region of western India and proposes the use of SB to complement KMC by keeping the baby warm. The strategy may be to place the baby in an SB when the baby is not in KMC.

### The need to complement KMC

Between 1990 and 2015, the global under-five mortality rate dropped by 53%, i.e., from 94 to 41 deaths per 1,000. However, this decline was not enough to meet the global MDG4 target to reduce the under-five mortality rate by two-thirds. Neonatal deaths account for 44% of the world’s under-five child mortality rate, which is decreasing slowly [4]. Improving the care of preterm infants is an important issue since more than half of neonatal deaths globally occur in preterm infants. LBW is often the proxy for prematurity when there is difficulty in determining gestational age, especially in developing countries. Improving the quality of care of LBW infants figures prominently among the top 10 research priorities identified for reducing global neonatal mortality for preterm birth and LBW, as set by the UN’s Millennium Development Goal 4 [5]. The World Health Organization’s (WHO) new guidelines on interventions to improve preterm birth outcomes include a set of key interventions that comprises thermal care, safe oxygen use, and the use of surfactant to help infants breathe more easily [6].

KMC, with skin-to-skin contact (SSC) as its core component, is a safe, effective and affordable method for keeping preterm babies warm and is associated with 36% lower mortality among LBW newborns in resource-limited settings compared to conventional care [7]. Therefore, KMC should be an integral component of neonatal care in such areas. The WHO has endorsed KMC for stabilizing newborns in health facilities in both high-income and low-resource settings [8]. However, despite its advantages, the implementation of KMC has been limited, which is largely because of a lack of help in KMC practice and with other obligations that need to be met. Identifying alternatives to KMC for keeping infants warm in a community setting has, therefore, emerged as one of the top 10 research priorities to reduce global mortality of preterm birth and LBW neonates [5].

Globally, three million newborns die every year. Among these, nearly 2 million/year die in the first week after their birth, and significantly, 1 million/year die on the first day of their birth, making it the riskiest day for them [9]. To reduce the number of neonatal deaths, KMC should be available for LBW infants on the first day of life. However, in most settings, promoting SSC for 24 h a day is not feasible due to various barriers in the early days of life [10]. In a study of babies that were given SSC, it was observed that in as many as 94 infants (71%), a peripheral body temperature below 32 °C was recorded at least once, and in 29 of those, it dropped below 30 °C when the infants were not receiving SSC [11]. To prevent these episodes of cold stress/hypothermia, a baby should be in an SB when not receiving SSC. “Placing a baby in an SB when not receiving SSC may prevent these episodes of cold stress/hypothermia.”

**Fig. 1:**
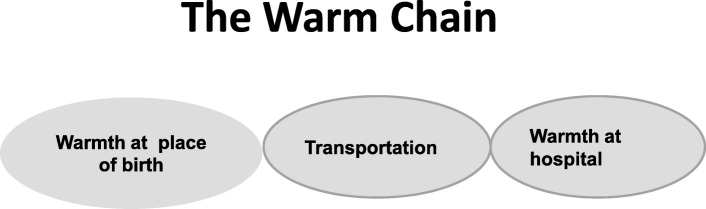
The Warm Chain

SBs can also help to strengthen the referral system by functioning as “transport incubators.” Referral transfers may be necessary from a home setting to a primary health center (PHC) or from a PHC to a first referral unit (FRU) or a district hospital [12, 13]. During an inter-hospital transfer in SB, the administration of oxygen, feeding and intravenous (IV) medications is not interrupted [14, 15]. Thus, an SB can complement KMC by strengthening the “warm chain” from the place of birth, during transportation and at the place of subsequent care (Fig. 1).

### About the device

**Fig. 2:**
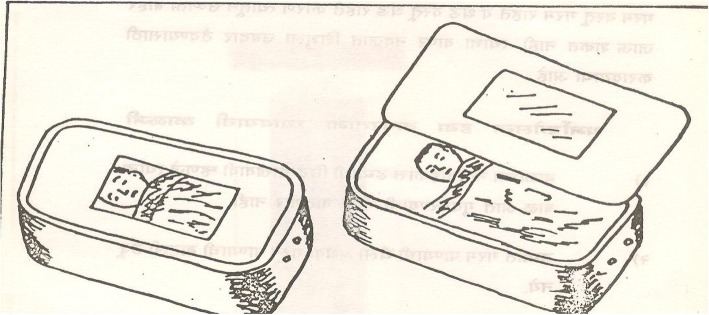
A baby in Styrofoam box at home

Styrofoam (expanded polystyrene) has good insulation properties, has low thermal conductivity and is chemically inert. Heat loss by convection, evaporation and radiation can also be prevented in an SB. Styrofoam has zero nutritional value, so it is resistant to bacterial growth and can be reused after cleaning with soap and water. A commercially available SB costs only $2.25 and is affordable. An SB that measures 50 × 36 × 25 cm and weighs 500 g appears to be appropriate for newborn care. The wall thickness and the lid thickness are 2.5 and 3.5 cm, respectively. Four breathing holes are made, with two on each of the narrower sides in coaxial planes, 8 cm apart, 2 cm in diameter, and 8 cm above the base. An (optional) observation window (18 × 15 cm) may be carved out in the lid and a transparent polythene sheet may be fixed in place by an adhesive tape. The clothing worn by an infant in the box would depend on the season and the size of the baby (Fig 2).

### Details of the studies performed

**Fig. 3:**
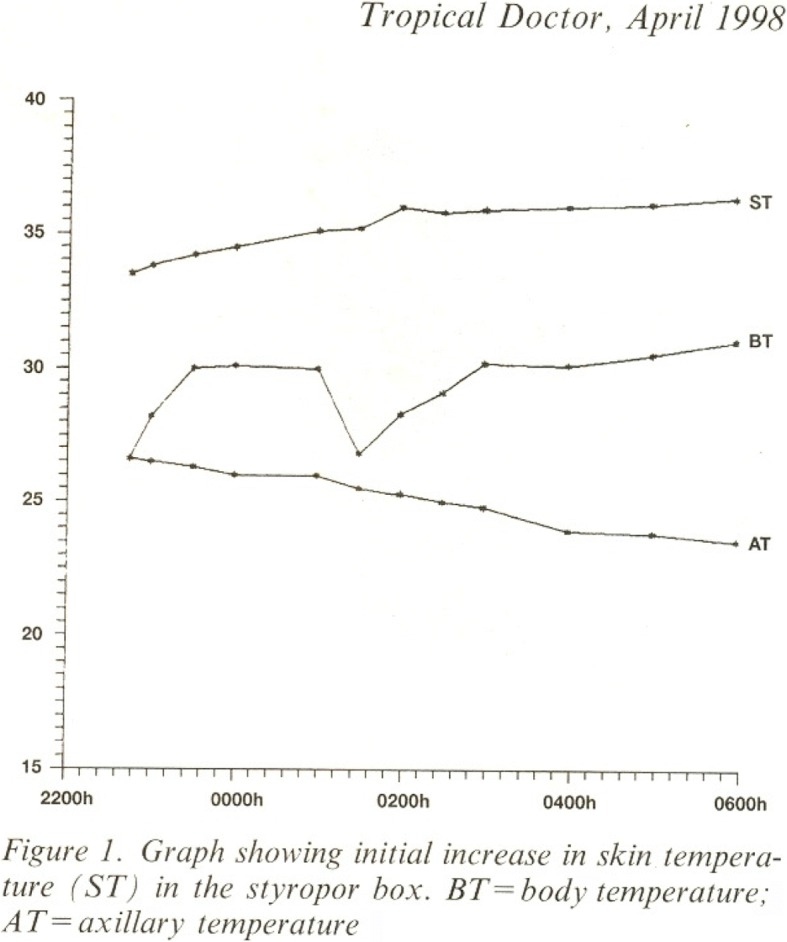
Graph showing box, skin and atmospheric temperature during study period

1. SBs as home incubators in rural areas: It is case series (*n* = 6), that described the in-home care of six very low birth weight babies, (foot length < 6.5 cm). The parents declined hospital care due to the sowing season [16]. However, they agreed to in-home care. The distance between their homes and the PHC headquarters ranged between 1 and 7 km. The babies had no birth asphyxia or respiratory distress. The in-home care began soon after birth. They received colostrum and sugar water for the first 2–3 days. Afterward, they received only expressed breast milk. The infants were kept in an SB wrapped in two layers of a soft cotton sari. The infants were removed from the SB periodically for feeding. A baby that developed cold feet was rewarmed by providing warm fomentation [17]. Auxiliary nurse midwives, link workers (health workers chosen from the community), and traditional birth attendants (TBA) supervised the in-home care. All six infants survived till an available follow up of 3 months.
2. SBs for infants discharged from the hospital: As a proof of concept, LBW babies (*n* = 7) were studied to see if they were warm in SBs at the home setting after discharge from the hospital [18]. The temperatures were recorded at home from 10 pm to 6 am. Ambient air temperature (AT), air temperature in the box (BT) and skin (plantar sole) temperature (ST) were recorded every 30 min at home from 10 pm to 6 am. The difference between the AT and the BT was statistically significant, indicating the ability of the SB to achieve a higher temperature by effectively retaining the metabolic heat liberated by the infant (Fig. 3). Second, the difference in the ST was statistically significant when the initial temperature was low, indicating that the infant was rewarmed in the SB.
3. SBs for transporting infants from the home setting to the PHC: In a pilot study conducted, an SB was kept at each hamlet of a village to ensure immediate transportation of VLBW infants born with foot length < 6.5 cm for hospital care [13]. This initiative was a part of the Rural Neonatal Care Project, Dahanu, which was started by the state government. The SBs were kept at a strategic location for ready availability, either at the residence of a TBA or of a link worker. The referrals from the home setting to the PHC and from the PHC to the FRU steadily increased [12] (Table 1), which indicates that acceptance of this device by the community was positive. Most of these infants were transported on the first day of life.
4. SBs for inter-hospital transfers: In a feasibility study (*n* = 32) high-risk newborns with respiratory distress (19 infants), preterm (9), shock (4), sepsis (4), and surgical problems (4) that required continuation of special care or surgical intervention were transported from Cama Hospital to J.J. Hospital, Mumbai. The transportation time was 30 min on average. Oxygen was bubbled in through the breathing hole of the SB. In critically ill infants, the inotrope infusion was continued during transport by passing the IV tubing through one of the breathing holes. Infants on enteral feeds received their feed 30–45 min prior to transportation [14]. During the inter-hospital transfers, none of the babies became hypothermic, failed to maintain oxygen saturation or developed signs of hypoglycemia. The heart and respiratory rates of each baby remained within the acceptable ranges.
5. Oxygen administration in SB: In a pilot study, as a step-down therapy from head boxes, oxygen from a 660-l cylinder was administered to preterm infants (no = 8) in SBs [15] through one of the ventilation holes in each SB at 0.5 L/min. The oxygen concentration in the box was recorded at 5, 10, 15 and 30 min using an oxygen analyzer (Oxydig, Draggers). The measurements commenced 30 min after a feed. The oxygen saturations were recorded using a pulse oximeter (Systems Biomedical). The monitoring cords were inserted through other ventilation holes.

**Table 1:** Year-wise referrals to PHC^a^ for special care

Year	Home to PHC	PHC to First Referral Unit
1988	10	3
1989	14	3
1990	20	4

^a^*PHC* Primary Health Centre

The differences between the oxygen concentrations in the box at different intervals were compared (t test). The intervals chosen for comparison were: 0 vs. 5 min, 5 vs. 10 min, 10 vs. 15 min, and 15 vs. 30 min. The oxygen concentration in the box increased steadily at each interval from 0 to 15 min. Statistically significant differences were observed between 0 and 5 min, 5 and 10 min, and 10 and15 minutes. There was no significant difference in the oxygen concentration between 15 and 30 min. These findings suggest that oxygen concentration steadily and significantly rose between 0 and 15 min and was maintained thereafter.

### Comments

KMC offers many more benefits besides thermal protection, notably a successful initiation and maintenance of lactation. However, KMC offered for less than 7 h a day in the first 2 days of life does not offer substantial health benefits. In a trial of community-initiated KMC with 1565 mother-infant pairs, only 23.8% practiced SSC for more than 7 h/day in the first 48 h of life, and the average number of hours of SSC during days 3–7 of life was 2.7 ± 3.4 h [19]. A survey of 46 mothers of preterm infants who had been trained on KMC in a hospital in Andhra Pradesh, India, found that only 6.5% of mothers felt that providing KMC for 12 h/day or greater was feasible, whereas 52% of mothers felt that only 1 h/day was practical [20]. Lack of help with KMC practice and other obligations ranked among the top five barriers to KMC practice at home across all publications in lower- and middle-income countries (LMIC) [21]. Conversely, support from family, friends, and other mothers emerged as the third-highest-ranked enabler to KMC practice [21]. This support meant taking turns holding the infant in KMC to give the mother a break from the practice or taking care of other tasks that the mother otherwise would have had to address, including childcare and housekeeping.

## Conclusion

It is a matter of concern that the national and global coverage of KMC remains low. A premature baby should be in an SB when not in KMC for thermal protection at least. “Placing a premature baby in a SB when not in KMC should be considered for thermal protection at least.” However, large, well-designed studies are required to confirm the safety and efficacy of SBs to keep premature babies warm, reduce early neonatal mortality, and optimize/complement KMC in resource-limited set-ups.
